# Role of Periostin and Nuclear Factor-κB Interplay in the Development of Diabetic Nephropathy

**DOI:** 10.3390/cells11142212

**Published:** 2022-07-15

**Authors:** Lilia Abbad, Niki Prakoura, Arthur Michon, Rym Chalghoumi, Simone Reichelt-Wurm, Miriam C. Banas, Christos Chatziantoniou

**Affiliations:** 1 Unite Mixte de Recherche Scientific 1155, Institut National de la Sante et de la Recherche Medicale, Tenon Hospital, 75020 Paris, France; lilia.abbad@inserm.fr (L.A.); niki.prakoura@gmail.com (N.P.); art.michon@gmail.com (A.M.); rym.chalghoumi@gmail.com (R.C.); 2 Faculty of Medicine, Sorbonne University, 75020 Paris, France; 3 Department of Nephrology, University Hospital Regensburg, D-93053 Regensburg, Germany; simone.reichelt-wurm@klinik.uni-regensburg.de (S.R.-W.); miriam.banas@klinik.uni-regensburg.de (M.C.B.)

**Keywords:** periostin, chronic kidney disease, NF-κB, inflammation, fibrosis, diabetic mice

## Abstract

Diabetic nephropathy (DN) remains the most common reason for end-stage renal disease and a leading cause of kidney replacement therapy. Multifactorial pathophysiological mechanisms underlie the development of DN. Among the signalling pathways involved, nuclear factor-κB (NF-κB) plays a key role in pathogenesis triggering inflammation, oxidative stress and fibrosis. Recent evidence shows that periostin, a matricellular protein, is involved in the development of renal glomerular diseases through interaction with NF-κB signalling. The aim of the present study is to investigate the contribution of periostin and its interaction with NF-κB in DN development. To this end, we used the BTBR ob/ob mice model of diabetes type 2, and we applied transcriptomic analysis, immunostaining and methods quantifying protein and mRNA expressions. We found that increased periostin expression was correlated with decreased renal function, advanced stage renal damage and fibrosis, and NF-κB activation. Subsequently, we identified novel pathways and genes regulated by the NF-κB-periostin interaction which are involved in the mechanisms of progression of DN. Some of these genes, such as FGF1 and GDF15, have the potential to be new biomarkers and/or targets for the therapy of DN.

## 1. Introduction

Diabetic nephropathy (DN) is a chronic progressive disorder and the most common leading cause of renal failure worldwide. DN is a multifactorial disease that arises as a complication of type 1 and 2 diabetes. Although not all diabetic patients develop nephropathy, type 2 diabetes remains the primary cause of DN and its prevalence is estimated to affect more than 642 million people by 2040, which corresponds to a 50% increase in less than 20 years and represents 8–10% of the world’s population [1]. Multiple pathophysiological processes are associated with the progression of DN, including glomerular hyperfiltration, mesangial and tubular hypertrophy, podocyte injury and albuminuria [2]. These pathological features are associated with endothelial dysfunction, inflammation and fibrosis. Activation of the NF-κB pathway is one of the major drivers of the inflammatory processes associated with DN [3,4].

Although significant progress has been made over the past few decades in the treatment of DN, the standard therapeutic approaches, mainly based on the control of blood pressure, reduction of blood glucose, and blockade of the renin–angiotensin–aldosterone system are only partially effective and associated with important side effects [5,6]. More recently, glucose-lowering oral agents such as sodium–glucose cotransporter 2 (SGLT2) inhibitors have been proposed as a new promising therapeutic strategy [7,8]. These approaches will certainly improve DN healthcare and delay disease progression but will have limited efficacy in advanced stages when the only therapeutic options are dialysis or transplantation. Therefore, studying the mechanisms of DN pathogenesis remains an important medical need. Standard renal biomarkers of renal function, such as albuminuria, serum creatinine or glomerular filtration rate, are currently measured to monitor the progression of DN [9]. However, these parameters are insufficient to determine renal tissue injury or predict clinical outcomes, and they need to be associated with additional specific markers.

Emerging data indicate periostin as a novel marker and mediator of chronic kidney disease CKD [10,11]. Periostin is a 90 kDa matricellular protein which binds to integrins to stabilize the extracellular matrix during development. It is de novo activated during several pathological processes to promote cell proliferation, migration, differentiation, tissue fibrosis and inflammation [12,13,14,15,16]. Periostin is strongly up-regulated in several renal disease models and kidney biopsy specimens. We and other investigators found that increased periostin expression was strongly correlated with the degree of renal lesions and the decline of renal function [17,18,19]. Subsequent studies have shown that periostin, in addition to being a marker, is an active player in the progression of kidney disease. Indeed, mice lacking periostin are protected against the development of kidney disease in several models of CKD. In addition, periostin inhibition by antisense oligonucleotides delays and in some cases reverses the progression of CKD in hypertensive and glomerulonephritis animal models [19,20]. Interestingly, the activation of periostin in these models is induced mainly through the NF-κB signalling [20]. In humans, periostin is highly expressed in biopsies from patients with different renal diseases, including diabetic nephropathy [17,21]. Specifically, periostin levels are increased both in the renal tissue and in the urine of patients with type 2 diabetes, as well its levels are positively correlated with ageing, high albuminuria and the decline of renal function. Of note, targeting periostin with periostin-binding DNA aptamer treatment attenuates renal fibrosis in an experimental DN model [21].

In the present study, we investigated whether periostin is a mediator of renal disease in type 2 diabetes. Periostin is studied in the experimental model of DN (ob/ob) mice. By unbiased transcriptomic analysis, we compared the different expression levels of periostin in the kidney in order to identify new signalling networks regulated by periostin during DN progression. 

## 2. Materials and Methods

### 2.1. Mice

Six-week-old male BTBR WT controls and BTBR ob/ob mice were purchased from Jackson Laboratories (Bar Harbor, ME, USA). Animals were housed in a specific pathogen-free facility at a constant temperature with free access to water and food. Mice were euthanized at 12 weeks or 18 weeks. Blood was collected and kidneys were isolated and then immediately fixed in formalin. All procedures regarding animal experimentation were in accordance with the European Union Guidelines for the Care and Use of Laboratory Animals and approved by the local ethics committee of the National Institute for Health and Medical Research (Institut National de la Santé et de la Recherche Médicale, INSERM, Paris, France).

### 2.2. Biochemical Parameters

Blood glucose levels and plasma cholesterol were measured at biochemical platforms of CRC. Urines were collected from animals at 18 weeks and microalbumin excretion was measured in an Olympus AU400 biochemical analyser (Olympus, Rungis, France) at biochemical platforms of CRI and calculated by normalizing the albuminuria concentration with the creatinine level.

### 2.3. Histological Coloration and Immunostaining

#### 2.3.1. Histology

Half kidneys were fixed in 4% formalin solution and embedded in paraffin. Sections of 4 μM were stained with Periodic Acid Shiff (PAS) and Masson’s trichrome coloration. 

#### 2.3.2. Immunohistochemistry

Tissue was deparaffinized and antigen retrieval was made with 10 mM citric acid pH 6.1 (target retrieval solution, Agilent, Les Ulis, France) or with Tris-EDTA pH8 (Euromedex, Souffelweyersheim, France) at 95 °C. Endogenous peroxidase activity was blocked by peroxidase-blocking solution (Dako Agilent, Les Ulis, France). Sections were blocked and permeabilized with PBS with 10% BSA and 0.1% triton. The sections were then incubated with specific primary antibodies against periostin (R&D Systems, Lille, France). Secondary antibodies coupled with HRP were used for detection (Histofine^®^, Brignais, France).

#### 2.3.3. Immunofluorescence

The staining was performed on paraffin-embedded tissue in a similar manner to immunohistochemistry. Then the sections were incubated with primary antibodies for: periostin (R&D Systems, Lille, France), α-SMA (Abcam, Paris, France), FGF1 (Invitrogen, Les Ulis, France) and GDF15 (Invitrogen, Les Ulis, France). Alexa fluor (Invitrogen, Les Ulis, France) secondary antibodies were used for detection. Images were obtained with an OlympusIX83 microscope at 100×, 200× or ×400 magnification.

### 2.4. RNA Sequencing

RNA sequencing analysis was carried out for 12 (4 for each subgroup: low, medium and high, respectively) mouse kidney RNA samples and sent to AtlasBiolabs (Berlin, Germany) for processing. The RNA was adjusted to 100 ng RNA/ul and enriched by polyA affinity kits before sequencing. Subsequently, the samples were sequenced on an Ilumina Nova sequencing device (“paired-end” analysis), with a sequencing depth of more than 30 million base pairs (bp) with an average sequence length of 136 bp. Raw data were imported into ArrayStudio (Qiagen, Les Ulis, France) Omicsoft software. The “RNA-Seq-Pipeline” was used to align the sequence fragment of each sample to a mouse genome model and a reference library mouse gene. Standard normalization was performed which gave values in FPKM (fragments per kilobase million) for the mRNA of each gene and isoform detected in the sample, indicating a normalized expression value. The identification of differentially expressed genes (DEG) was performed by a robust statistical algorithm in case of a low number of replicates and low variability, VOOM.

### 2.5. Total RNA Extraction and Quantitative Real-Time PCR

Total RNA from renal tissue was extracted using TRIzol reagent (Invitrogen). One microgram of total RNA was reverse-transcribed in cDNA. The quantitative RT-qPCR, was performed with the following primers with an SYBR Green kit (Roche, Boulogne-Billancourt, France) on a CFX96 RT-PCR Detection System (Bio-Rad, Marnes-la-Coquette, France). Analysis of relative gene expression was performed by using the (2^−ΔΔCT^) method and normalised by housekeeping genes (RPL32 and HPRT). Primer sequences are listed in Table 1.

### 2.6. Western Blot Analysis

Proteins were extracted from renal tissue using RIPA lysis buffer (Santa Cruz Biotechnology, Heidelberg, Germany) supplemented with a protease cocktail inhibitor, PMSF, and sodium orthovanadate. An equal amount of denaturing samples was loaded in SDS-PAGE (4–12% polyacrylamide gels, Invitrogen) and transferred to nitrocellulose membranes. Immunoblotting was then performed with the following antibodies: periostin (R&D Systems), nephrin (R&D Systems, Lille, France), α-SMA (Abcam, Paris, France), p65 (Abcam, Paris, France), p-p65 (Cell Signalling, Dellaertweg, The Netherlands), FGF1 (Invitrogen Les Ulis, France) and GDF15 (Invitrogen, Les Ulis, France). Proteins were detected using the chemiluminescent substrate (Clarity Western ECL, Bio-Rad Marnes-la-Coquette, France) and for imaging used ChemiDoc (MP imaging, Bio-Rad, Marnes-la-Coquette, France). The protein signals were quantified and normalised to the corresponding loading controls GAPDH (Sigma-Aldrich, St Quentin-Fallavier, France).

### 2.7. Statistical Analysis

Data were presented as means ± standard error of the mean (SEM) and the statistical comparison was performed using Prism Software 8 (GraphPad Software Inc., San Diego, CA, USA). The comparison was made by using the non-parametric Mann–Whitney, or by using analysis of variance (ANOVA) with one-way ANOVA followed by Bonferroni for multiple comparisons. Statistical significance was defined as *p* < 0.05.

## 3. Results

### 3.1. Renal Function and Structure in Diabetic Ob/Ob Mice

**Figure 1:**
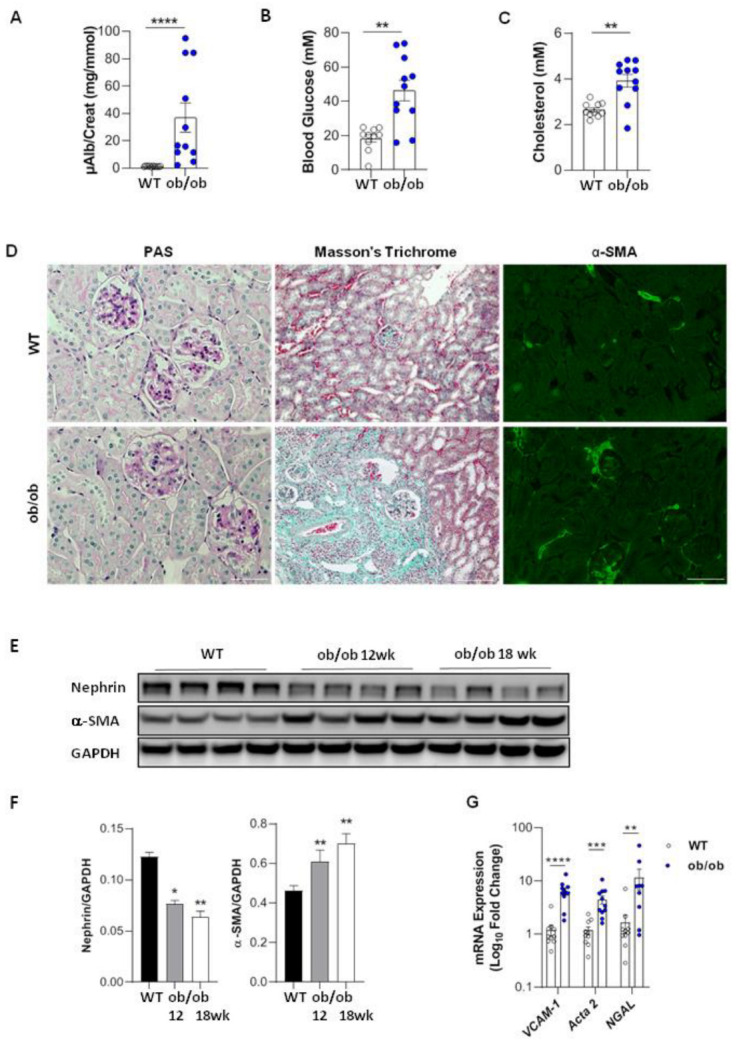
Renal function and structure in diabetic ob/ob mice. Microalbuminuria (µAlb)-creatinine ratio (**A**), blood glucose (**B**) and cholesterol (**C**), representative examples of Periodic Acid-Schiff (PAS), Masson’s trichrome coloration (×100), and α-SMA immunofluorescence staining (×200) (**D**), nephrin and α-SMA protein expressions by WB (**E**,**F**), and mRNA gene expressions of *VCAM-1, Acta2,* and *N-GAL* (**G**), in ob/ob mice. * *p* < 0.05, ** *p* < 0.01, *** *p* < 0.001, **** *p* < 0.0001 compared to WT controls. Bars = 50 µM (right) and 100 µM (middle, left). *n* = 10 WT mice vs. 11 ob/ob mice (**A**–**D**,**G**). In WB experiments, *n* = 4 mice per group, WT, and 12 weeks or 18 weeks old ob/ob mice (**E**,**F**).

Among the different models of DN we chose to use the BTBR ob/ob mutant mouse (ob/ob), this model mimics better than other models the development of type 2 DN seen in humans. At 18 weeks of age, the mice presented increased microalbuminuria and hyperglycemia and had elevated total cholesterol levels in plasma compared with age-matched BTBR WT controls (WT) (Figure 1A–C). These findings are in agreement with the renal phenotype in ob/ob mice described in previous studies [22]. In addition, ob/ob mice exhibited glomerular hypertrophy with a marked mesangial expansion, associated with glomerulosclerosis, focal and mild fibrosis (Figure 1D). Mesangial cell activation was confirmed by immunostaining for α-smooth muscle actin (α-SMA) (Figure 1D) and by increased *Acta2* mRNA and protein expression in the kidney tissue starting from 12 weeks of age (Figure 1E–G). Decreased protein expression of nephrin, a major component of the glomerular filtration barrier, suggested podocyte injury (Figure 1E,F). Furthermore, increased vascular cell adhesion molecule-1 (VCAM-1) and neutrophil gelatinase-associated lipocalin (NGAL) mRNA expression indicated the presence of kidney tubular cell stress and inflammation (Figure 1G). However, we observed that the degree of alterations in renal function and structure was heterogeneous within the group of age-matched ob/ob mice. As shown in Figure 1A–C, some ob/ob mice displayed normal levels of albuminuria, glycaemia or cholosterolaemia, whereas others had clearly developed DN. We have also noticed a mortality starting at 10 weeks of age which gradually increased with age to reach 25% at 18 weeks (Figure S1). For this reason, we established the age limit for our study at 18 weeks.

### 3.2. Periostin Is Associated with the Degree of Renal Injury in Diabetic Ob/Ob Mice

**Figure 2:**
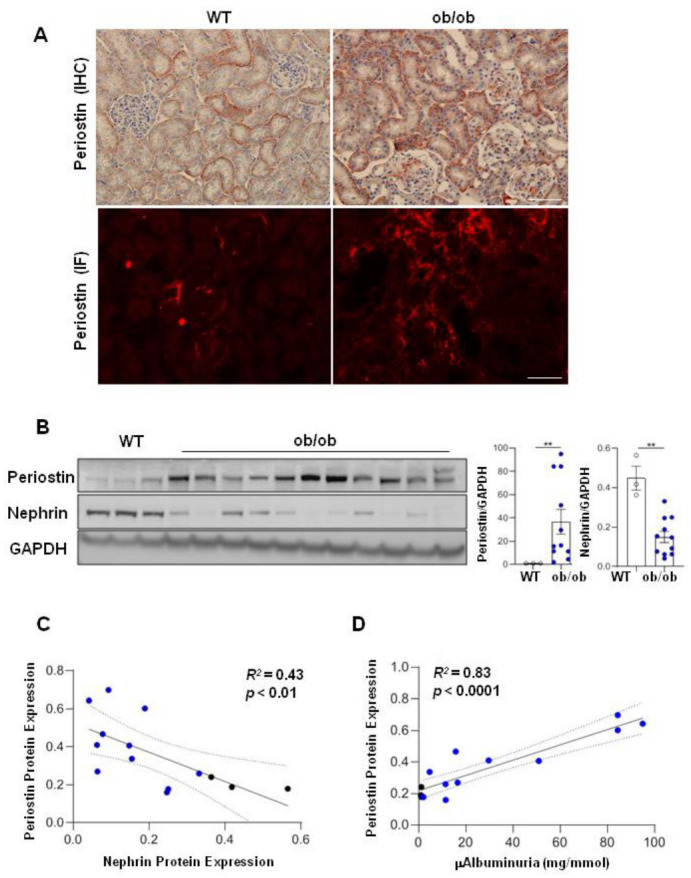
Periostin is associated with the degree of renal injury in diabetic ob/ob mice. Representative immunostaining of periostin tissue expression in kidneys by immunohistochemistry and immunofluorescence comparing WT and ob/ob mice (×200) (**A**). Periostin and nephrin protein expression in kidneys of WT (*n* = 3) and ob/ob mice (*n* = 11) (**B**). Correlations between periostin and nephrin (**C**) or µAlbuminuria (**D**). ** *p* < 0.01, compared to WT control. Bars = 50 µM.

In diabetic ob/ob mice periostin expression was increased in kidney tissues and mainly localized in tubulointerstitium (Figure 2A). We also observed periostin expression in the interstitial fibrotic zones, as well as in peri-glomerular areas. Labelling within glomeruli was limited to the fibrotic areas (Figure 2A). Some ob/ob mice showed strong staining of fibrosis and periostin whereas other age-matched ob/ob displayed negligible fibrosis and periostin staining (Figure S2). Quantification by Western blotting indicated that periostin expression was negatively correlated with nephrin and positively with micro-albuminuria (Figure 2C,D). Again, heterogeneity in the expressions of periostin and nephrin was observed within the age-matched group of ob/ob mice (Figure 2B). These data showed that periostin level correlates with the gravity of renal lesions, suggesting that periostin is a marker of renal injury of DN progression, as was demonstrated in other non-diabetic models of CKD [18].

### 3.3. High Periostin Levels Are Associated with an Activation of the NF-κB Pathway

**Figure 3:**
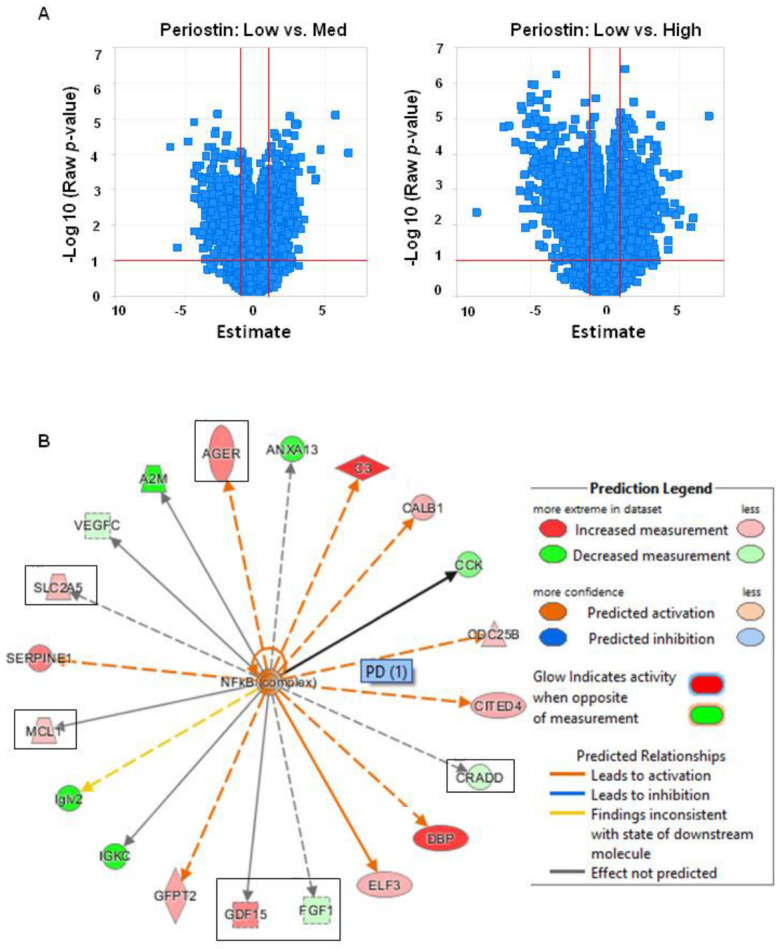
High periostin levels are associated with an activation of the NF-κB pathway. Volcano plots of differential gene regulation “low” versus “medium” and “low” versus “high” periostin expression. The log *p*-value for each gene is shown over the fold change in the expression (FKPM). The cut-off lines represent a *p*-value of 0.05 and a fold change of −2 or 2 (**A**). NF-κB mediated gene regulation (**B**).

We next sought to identify the pathways that could trigger periostin expression during DN. Given the heterogeneity observed between animals with respect to the severity of renal lesions and periostin expression, we decided to divide the mice into 3 subgroups based on microalbuminuria levels (which correlated with renal nephrin protein expression levels of periostin, as described in Figure 2C: “low” periostin expression <1 mg/mmol microalbuminuria” corresponding to the WT mice, “medium” periostin expression with <20 mg/mmol microalbuminuria and “high” periostin expression >20 mg/mmol of microalbuminuria” corresponding to ob/ob mice with mild and severe renal injury, respectively. We performed this type of classification because our purpose was to identify genes that play a role in the development of DN and are associated with periostin. A comparison between the samples from WT “low” versus ob/ob “medium” and WT “low” versus ob/ob “high” periostin expression revealed approximately 440 and 778 genes differentially regulated (DEG) with a corrected *p*-value below *p*(FDR) < 0.05 (FDR: false discovery rate). The gene expression differences between low vs. high were stronger than with low vs. medium (Figure 3A). This analysis allowed us to identify more than 6 pathways, with a significant activation or inhibition score and a significant corrected *p*-value. Thus, activation of pro-inflammatory (NF-κB, MAPK14, IL-33), anti-apoptotic (AKT1, NOTCH2) and pro-fibrotic (HSF1, AGT) signalling pathways were increased in “high” periostin diabetic mice (Table S1). We focused on the NF-κB pathway since we and other investigators have previously demonstrated that NF-κB activation promotes periostin expression in glomerulonephritis and also in a model of pulmonary fibrosis, respectively described [20,23]. Moreover, we found twenty-four genes whose variation in expression was found to be positively or negatively correlated with differences in periostin expression (Table S2). From this list, we first excluded genes whose expression is known to be associated with obesity, such as genes of carbohydrate (carboxylesterase 1G) or lipid metabolism (apoliprotein H, lipoprotein lipase), and selected six genes which are associated to the NF-κB pathway: fibroblast growth factor 1 (FGF1), growth differentiation factor 15 (GDF15), advanced glycation end products receptor (AGER), death domain-containing protein (CRADD), sodium–glucose cotransporters (SCL5A2) and myeloid leukaemia cell differentiation protein (MCL-1) (Figure 3B).

### 3.4. Periostin Mediates Renal Inflammation and Fibrosis through NF-κB by Repressing FGF1 and GDF15

**Figure 4:**
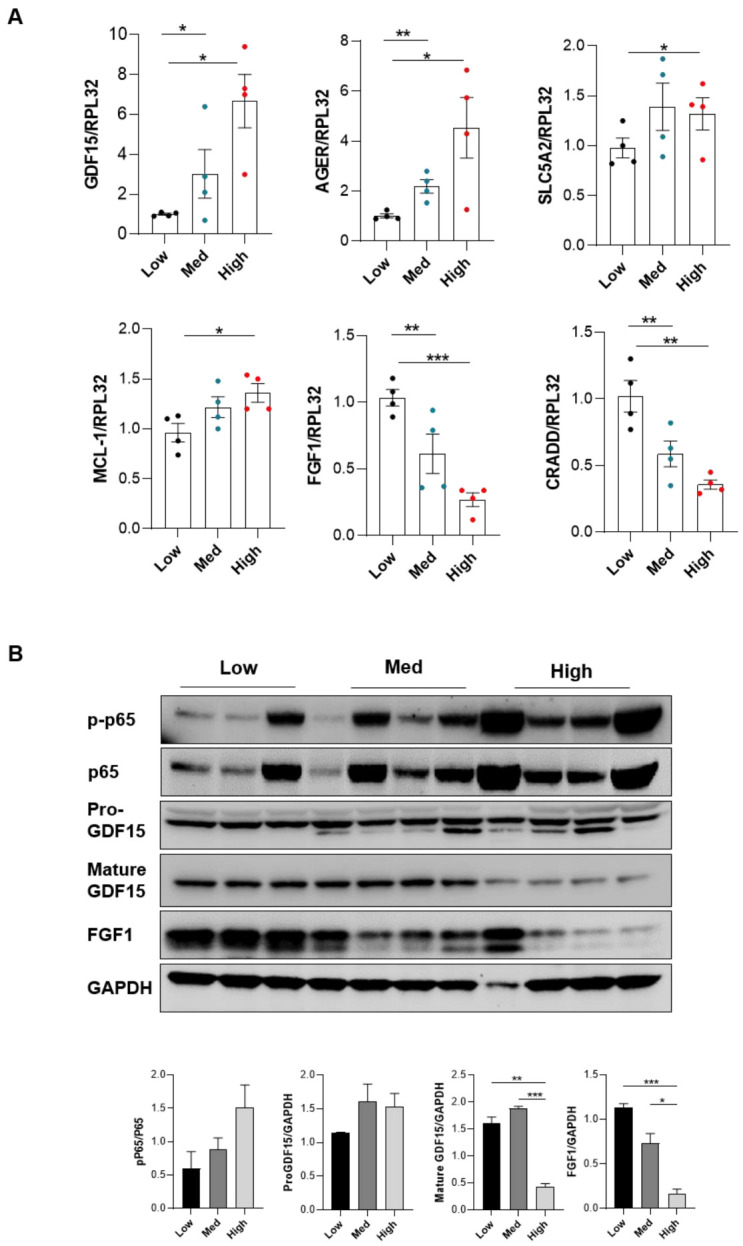
Periostin mediates renal inflammation and fibrosis through NF-κB by repressing FGF1 and GDF15. Gene expression of GDF15, AGER, SCL5A2, MCL1, FGF1 and CRADD according to periostin level (genes expression was normalised by housekeeping genes (RPL32 and HPRT) (**A**). Immunoblotting showed protein expression of p-p65, p65, Pro-GDF15, mature GDF15 and FGF1 in kidney mice with low, medium and high periostin expression (**B**). * *p* < 0.05, ** *p* < 0,01, *** *p* < 0.001.

**Figure 5:**
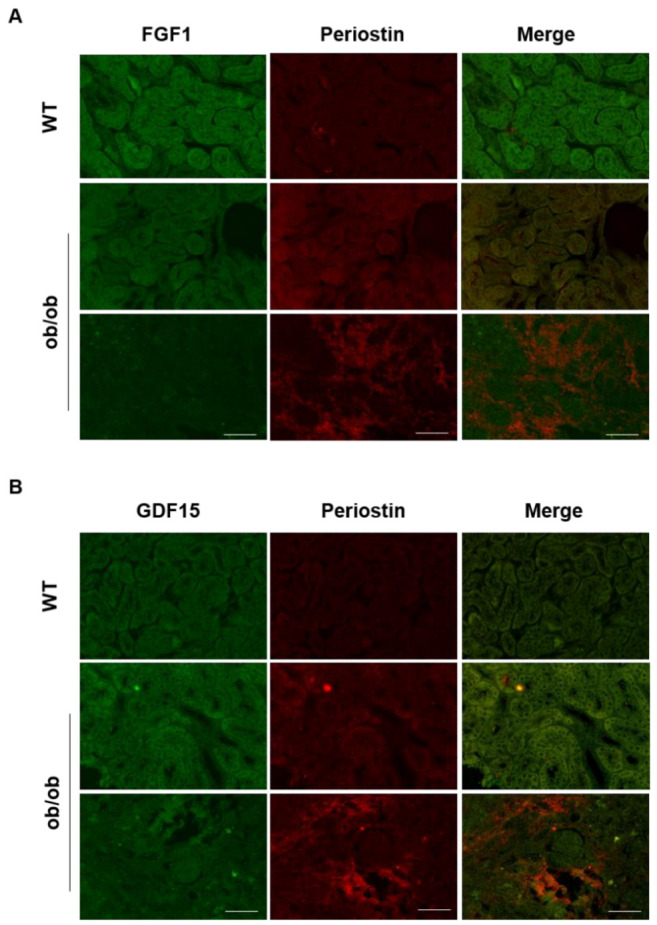
Periostin upregulation represses tubular FGF1 and GDF15 expression. Immunofluorescence of FGF1 and periostin (**A**). Immunofluorescence of GDF15 and periostin (×200) (**B**), Bars = 50 µM.

The differential expression of genes identified by RNAseq analysis (Figure S3) was confirmed by qPCR (Figure 4A). In particular, increased expression of GDF15, AGER, SCL5A2 and MCL-1 was observed in mice with increased expression of periostin. Conversely, lower expression of FGF1 and CRADD was associated with increased periostin expression. Other genes, including SCL5A2, MCL-1 and CRADD, were regulated by NF-κB independently of periostin variation, suggesting that these genes although mediated by the NF-κB pathway and associated with DN are not systematically associated with periostin. SCL5A2 also known as SGLT2, plays an important role in tubular apoptosis and reactive oxygen generation in DN [24]. MCL1 and CRADD both are involved in apoptosis and NF-κB signalling [25,26,27,28]. The involvement of AGER was confirmed by bioinformatics and validated by qPCR. Indeed, AGER activation promotes cell dysfunction and the release of pro-inflammatory cytokines by activating nuclear NF-κB [29]. To further validate the role of the NF-κB pathway in the ob/ob model, we evaluated the expression of the identified targets from kidney extracts of the same mice used for the RNAseq analysis (Figure 4B). NF-κB activation was studied by assessing the levels of p65 phosphorylation, one of the most abundant forms present in most cell types. Increased p65 levels were observed in ob/ob compared to WT mice. Furthermore, p65 phosphorylation was increased in mice with high periostin levels, suggesting a correlation between periostin and p65 activation. One sample from the group “high periostin” was removed for the analysis because of the low protein expression of our loading control GAPDH. An opposite trend was observed for FGF1 expression whose levels were decreased and inversely correlated with periostin expression in ob/ob compared to WT mice. In WT mice, FGF1 immunostaining was mainly found in tubule cells (Figure 5A). In contrast, FGF1 staining was decreased in ob/ob mice displaying moderate periostin expression, and was almost absent in ob/ob mice with high periostin expression, particularly in the areas of fibrosis where periostin predominates (Figure 5A). A similar trend was observed for the mature form of GDF15 whose protein expression was inversely associated with periostin levels, whereas the inactive form pro-GDF15 and its related mRNA were increased (Figure 4A,B). By immunostaining, GDF15 localisation seemed also to be dependent on the presence or absence of periostin (Figure 5B). GDF15 expression was tubular, cytoplasmic and diffuse in samples with low levels of periostin. Conversely, in fibrotic areas where periostin is strongly induced, GDF15 was weakly detected or even absent (Figure 5B). Thus, when periostin is highly expressed both FGF1 and GDF15 are undetectable, whereas tubular areas with intermediate periostin expression presented simultaneously expression with FGF1 or GDF15. These data suggest that periostin in DN might repress the expression of FGF1 and prevent GDF15 activation, favouring in this way the establishment of renal fibrosis.

## 4. Discussion

In an era where medicine is increasingly personalized for rapid diagnosis and therapy, the identification of early biomarkers of diabetic nephropathy would represent a considerable advance for patients and healthcare establishments. In the present study, we investigated the role of periostin and its association with the NF-κB pathway in a type 2 model of DN. Using transcriptomic analysis, histology, immunostaining and methods quantifying protein and mRNA expressions, we identified novel pathways and genes regulated by the NF-κB-periostin interaction and which are involved in the mechanisms of progression of DN. Some of these genes, such as GDF15 and FGF1, have the potential to be new biomarkers and/or targets for the therapy of DN.

Two preclinical models are commonly used to study diabetic nephropathy in mice. For type 1 diabetes, streptozotocin (STZ)-induced diabetes is a well-established model. For type 2 diabetes, the genetically predisposed leptin-deficient ob/ob or leptin receptor-deficient db/db mice recapitulate well the human metabolic syndrome [24]. In the STZ model, mice develop mild renal lesions, similarly to that observed in early-stage human DN. The db/db mice and the ob/ob mice present a nearly identical phenotype, however, DN develops more rapidly in ob/ob mice with lesions similar to advanced human DN such as glomerular mesangial expansion, increased albuminuria, inflammation and podocytes injury [22,30,31].

In our experimental conditions, we observed a certain degree of heterogeneity in the severity of the renal phenotype of ob/ob mice at the same age. In addition, ob/ob mice started to die from 10 weeks of age, differing from a previous report stating that mortality is observed from 22–24 weeks of age [22]. However, we noticed that periostin was associated with the degree of alterations of renal phenotype and we took advantage of the observed heterogeneous phenotype to stratify ob/ob mice into two groups: one characterized by an early stage of renal disease showing intermediate periostin expression, and the other with advanced renal stage disease and high periostin expression, and mice with low periostin expression corresponded to WT mice. Indeed, mice with interstitial fibrosis and increased microalbuminuria presented a strong periostin staining in areas with fibrosis and vessels; tubules were the most predominant location for periostin staining. It was previously reported that periostin is highly expressed in patients with diabetic nephropathy [21] as well in other human nephropathies, such as lupus [32], IgA nephropathy [33], autosomal dominant polycystic kidney disease [34], focal-segmental glomerulosclerosis, or membranous nephropathy [17]. The abnormal periostin expression was observed in the mesangium, tubular interstitium and sites of fibrosis. In the present study, periostin was mainly expressed in tubular epithelial cells, and peri-glomerular, while intra-glomerular expression was mesangial, weak and limited to sites of focal fibrosis. This finding is consistent with observations made on renal biopsies from DN patients [21,35]. Furthermore, periostin expression in kidneys of ob/ob mice correlated well with microalbuminuria, podocyte injury (evaluated by the decreased nephrin expression) and fibrosis. Similar results, correlating periostin expression with the extent of renal injury, were found in other models of CKD [20]. High periostin expression correlated with worsened renal outcomes including elevated serum creatinine, estimated glomerular filtration rate and the chronicity of renal pathology [17,18]. In addition, the increased levels of periostin in urine significantly correlated with ageing, high albuminuria and the decline of glomerular filtration rate (GFR) [35]. Periostin intraglomerular expression was weak and limited to focal fibrosis, an observation also made in human biopsies from patients with advanced diabetic nephropathy [35].

Another objective of our study was to investigate the molecular pathways associated with periostin expression during DN progression. To this end, we performed a transcriptomic analysis of renal tissues by comparing WT mice to ob/ob mice with medium or high periostin expression. We identified more than six signalling pathways as differentially regulated according to periostin expression, and for the scope of the present study, we decided to focus on the NF-κB pathway. Activation of NF-κB and nuclear translocation of the p65 subunit has been described in human DN [3] and in the STZ model, and this activation plays a critical role in the progression of renal dysfunction [36,37]. The specific mechanism(s) leading to NF-κB activation in DN is still unclear. Under pathological/inflammatory conditions NF-κB is activated by diverse stimuli, from Toll-like and antigen receptors to cytokine and growth factor receptors, and promotes the transcriptional regulation of genes encoding proteins involved in immune and inflammatory responses (cytokines, chemokines, growth factors, immune receptors, and adhesion molecules) that are associated with chronic inflammation, fibrosis, and tissue remodelling [23,38,39,40]. The NF-κB family protein consists of homodimers and heterodimers formed by five subunits (p65, c-rel, RelB, p50, and p52). The most common heterodimer, p65/p50, is present in most cell types and is retained in an inactive form in the cytoplasm, then released to the nucleus activating the NF-κB signalling pathways [41,42]. Therefore, we explored the expression pattern of p65 in our experimental conditions. ob/ob mice displayed increased p65 protein expression compared to WT mice. Since phosphorylation plays a critical step in the activation of NF-κB, we measured p65 phosphorylation levels and we found that they positively correlated with high periostin expression, suggesting that the activation of NF-κB through p65/p50 could promote periostin expression during DN. This finding is in accordance with our previous study showing that NF-κB-induced periostin promotes renal injury in glomerulonephritis [20]. In this previous study, we found that the promoter of periostin contains three cis response elements to p65 and that binding of p65 in these elements was the strongest inducer of periostin transcription in cultured cells. Furthermore upon inflammatory aggression in vivo p65 binds to the periostin promotor and induces early activation of periostin gene transcription [20]. Altogether these data suggest that the NF-κB-periostin association is a common feature of CKD, at least in experimental models.

Among the twenty-four genes regulated by the NF-κB pathway that correlated with high expression of periostin, we chose to study the potential contribution of FGF1 and GDF15. Both have been described previously to have a link with NF-κB. For instance, FGF1 has been previously shown to inhibit the NF-κB pathway in experimental diabetic nephropathy [43], whereas GDF15–mediated inhibition of NF-κB signalling reduces infiltration of macrophages and arrests the pro-apoptotic activity in early tumour development [44]. In our study, we observed a decrease in mRNA and protein expression of FGF1 in the kidneys of ob/ob mice. This decrease was inversely and strongly correlated with periostin expression. These results are consistent with previous studies reporting decreased expression of FGF1 in another diabetic model (db/db) [43]. FGF1 has been reported to ameliorate diabetic nephropathy by an anti-inflammatory and antioxidant stress-induced mechanism. Interestingly, recombinant FGF1 injection significantly suppresses renal inflammation (cytokines, macrophage infiltration), glomerular and tubular damage, and renal dysfunction in both type 1 STZ and type 2 db/db diabetic mice [43,45]. FGF1 was included in the list of downregulated genes and related to mesangial proliferation and proteinuria [46] in gene expression profiling analysis from healthy and DN patient glomeruli. FGF1 is widely expressed in developing and mature tissues and regulates numerous biological activities. In our study, FGF1 was clearly expressed in tubular cells of WT mice, while dual labelling of FGF1 and periostin in fibrotic areas revealed decreased FGF1 expression concomitant to strong labelling of periostin. A similar inverse interaction between FGF1 and periostin has been observed in breast cancer where FGF1 repressed periostin expression through a PKC-dependent pathway [47]. Moreover, both FGF1 and periostin can directly bind cell-surface integrin αvβ3, suggesting that both can compete for the same receptor [48,49,50]. Taken together these results show that FGF1 can be an interesting therapy approach to mitigate the complications of DN.

Another finding of our study is the role of NF-κB/periostin in GDF15 activation. GDF15, also known as MIC-1, is a member of the transforming growth factor beta family [51] and is involved in the regulation of inflammation, apoptosis, cell growth and tissue repair [52]. GDF15 is produced as a pro-peptide form (≈40 kDa) which is processed to a mature dimeric form (≈25 kD) after cleavage of an N-terminus sequence [53]. Under normal conditions, GDF15 is expressed in several organs and up-regulated in response to injury [54]. In our study, mRNA/pro-peptide form of GDF15 and the protein expression of pro-GDF15 form were positively correlated with increased periostin levels and p65 activation, likely associated with the inflammatory response and renal damages in DN. As we have observed in our diabetic mice, GDF15 is expressed mainly in tubular cells in patients with CKD [55] and the urinary GDF15 levels and plasma levels are associated with kidney histology lesions in patients with CKD, including DN [55,56,57]. In GDF15 knock-out mice, it was observed that an increase in renal tissue damage in the STZ and db/db models was associated with an increase in tubular and interstitial injury. Moreover, an anti-fibrotic effect has been attributed to GDF15 by the blockage of the TGF-β receptor and the N-Myc signalling pathways in a ureteral obstruction model after GDF15 administration [55]. All these data suggest an anti-fibrotic effect of GDF15 and therefore the increase in pro-GDF15 observed in our diabetic mice could be considered as a marker and also as a protective reaction to limit the progression of DN. We think that the protective effect of GDF15 could be counteracted by the activation of the NF-κB/periostin axis in diabetic ob/ob mice during DN progression since the expression levels of the mature form of GDF15 were found to decrease in the kidneys of mice with advanced renal disease, high levels of periostin and activated p65. Furthermore, GDF15 expression was negligible in periostin-expressing fibrotic areas. This could suggest that the NF-κB/periostin axis might play a role in preventing the maturation of GDF15 into an active form, and/or in regulating its degradation in order to inhibit the anti-inflammatory and anti-fibrotic effect of GDF15. Due to their anti-inflammatory and antioxidant properties, both FGF1 and GDF15 targeting could be an alternative approach to limit DN pathology. There are several questions that need to be further addressed in order to understand the mechanism by which FGF1 and the active form of GDF15 are regulated during DN progression. At this stage we cannot predict which of the two proteins would be more effective, this is a new line of research to explore in follow-up studies. 

## 5. Conclusions

The present study suggests that periostin is an important marker reflecting renal damage and fibrosis in DN as well and that periostin regulation was dependent on the activation of the NF-κB pathway via the p65 subunit. The NF-κB-periostin axis could be the cause of the downward regulation of FGF1 expression and the activation of the mature form of GDF15. These two proteins are also being considered to be potential markers of DN progression. It would be interesting to explore the association of the NF-κB-periostin axis with FGF1 and GDF15 in urine and tissue of DN patients and to correlate their levels with disease stages. This would allow a better understanding of the mechanisms of DN progression, and establish patient profiles for better treatment. In addition, promoting treatments that will specifically activate FGF1 expression or GDF15 maturation would be a future therapeutic approach to limit DN progression.

## Figures and Tables

**Table 1:** List of sequences of the primers used in the RT-qPCR experiments.

	Forward	Reverse
*ACTA2*	GACACCACCCACCCAGAGT	ACATAGCTGGAGCAGCGTCT
*AGER*	GCCACTGGAATTGTCGATGAGG	GCTGTGAGTTCAGAGGCAGGAT
*CRADD*	AAGGCGAGAGAGGAAGTCACAG	AGCTGGTTAATCTGCTGGTCTGA
*FGF1*	CTGTGACAGCGCAAAGGCTG	TGGTAGGCTCAGCACTGAAGAA
*GDFF15*	CATAGGGGTGACTTGCCTGAA	CCCCACAGAACATGTGATGGA
*HPRT*	GGAGCGGTAGCACCTCCT	CTGGTTCATCATCGCTAATCAC
*KIM*	CCAACATCAATCAGAGTCTCTACC	TGTCTCATGGGGACAAAATG
*MCL1*	AGCTTCATCGAACCATTAGCAGAA	CCTTCTAGGTCCTGTACGTGGA
*RPL32*	GCTGCCATCTGTTTTACGG	TGACTGGTGCCTGATGAACT
*SLC2A5*	ATCGCTGCCTTTGGCTCATCCT	AGCAGCGTCAAGGTGAAGGACT
*VCAM1*	TGGTGAAATGGAATCTGAACC	CCCAGATGGTGGTTTCCTT

## Data Availability

Not applicable.

## Supplementary Materials

The following supporting information can be downloaded at: https://www.mdpi.com/article/10.3390/cells11142212/s1, Figure S1: Survival curve of WT and ob/ob mice; Figure S2: Periostin immunostaining in ob/ob mice fibrosis and with fibrosis; Figure S3: Regulation of FGF1, GDF15, AGER, CRADD, SCL5A2, and MCL1 with increasing periostin by RNAseq analysis; Table S1: Signalling pathways regulated in “high” periostin DN mice; Table S2: List of genes associated with periostin-NF-κB.

## References

[1] Ogurtsova K., da Rocha Fernandes J.D., Huang Y., Linnenkamp U., Guariguata L., Cho N.H., Cavan D., Shaw J.E., Makaroff L.E. (2017). IDF Diabetes Atlas: Global Estimates for the Prevalence of Diabetes for 2015 and 2040. Diabetes Res. Clin. Pract..

[2] Mirzaei M., Rahmaninan M., Mirzaei M., Nadjarzadeh A., Dehghani tafti A.A. (2020). Epidemiology of Diabetes Mellitus, Pre-Diabetes, Undiagnosed and Uncontrolled Diabetes in Central Iran: Results from Yazd Health Study. BMC Public Health.

[3] Mezzano S., Aros C., Droguett A., Burgos M.E., Ardiles L., Flores C., Schneider H., Ruiz-Ortega M., Egido J. (2004). NF-κB Activation and Overexpression of Regulated Genes in Human Diabetic Nephropathy. Nephrol. Dial. Transplant..

[4] Schmid H., Boucherot A., Yasuda Y., Henger A., Brunner B., Eichinger F., Nitsche A., Kiss E., Bleich M., Gröne H.-J. (2006). Modular Activation of Nuclear Factor-KappaB Transcriptional Programs in Human Diabetic Nephropathy. Diabetes.

[5] Arendse L.B., Danser A.H.J., Poglitsch M., Touyz R.M., Burnett J.C., Llorens-Cortes C., Ehlers M.R., Sturrock E.D. (2019). Novel Therapeutic Approaches Targeting the Renin-Angiotensin System and Associated Peptides in Hypertension and Heart Failure. Pharmacol. Rev..

[6] Banerjee D., Winocour P., Chowdhury T.A., De P., Wahba M., Montero R., Fogarty D., Frankel A.H., Karalliedde J., Mark P.B. (2022). Management of Hypertension and Renin-Angiotensin-Aldosterone System Blockade in Adults with Diabetic Kidney Disease: Association of British Clinical Diabetologists and the Renal Association UK Guideline Update 2021. BMC Nephrol..

[7] Choi C.-I. (2016). Sodium-Glucose Cotransporter 2 (SGLT2) Inhibitors from Natural Products: Discovery of Next-Generation Antihyperglycemic Agents. Molecules.

[8] Nair S., Wilding J.P.H. (2010). Sodium Glucose Cotransporter 2 Inhibitors as a New Treatment for Diabetes Mellitus. J. Clin. Endocrinol. Metab..

[9] Currie G., McKay G., Delles C. (2014). Biomarkers in Diabetic Nephropathy: Present and Future. World J. Diabetes.

[10] Turczyn A., Pańczyk-Tomaszewska M. (2021). The Role of Periostin in Kidney Diseases. Cent. Eur. J. Immunol..

[11] Jia Y., Yu Y., Li H.J. (2020). The Research Status and Prospect of Periostin in Chronic Kidney Disease. Ren. Fail..

[12] Snider P., Standley K.N., Wang J., Azhar M., Doetschman T., Conway S.J. (2009). Origin of Cardiac Fibroblasts and the Role of Periostin. Circ. Res..

[13] Kudo A. (2011). Periostin in Fibrillogenesis for Tissue Regeneration: Periostin Actions inside and Outside the Cell. Cell. Mol. Life Sci..

[14] Cobo T., Viloria C.G., Solares L., Fontanil T., González-Chamorro E., De Carlos F., Cobo J., Cal S., Obaya A.J. (2016). Role of Periostin in Adhesion and Migration of Bone Remodeling Cells. PLoS ONE.

[15] Masuoka M., Shiraishi H., Ohta S., Suzuki S., Arima K., Aoki S., Toda S., Inagaki N., Kurihara Y., Hayashida S. (2012). Periostin Promotes Chronic Allergic Inflammation in Response to Th2 Cytokines. J. Clin. Investig..

[16] Sözmen M., Devrim A.K., Kabak Y.B., Devrim T. (2019). Periostin Alters Transcriptional Profile in a Rat Model of Isoproterenol-Induced Cardiotoxicity. Hum. Exp. Toxicol..

[17] Sen K., Lindenmeyer M.T., Gaspert A., Eichinger F., Neusser M.A., Kretzler M., Segerer S., Cohen C.D. (2011). Periostin Is Induced in Glomerular Injury and Expressed de Novo in Interstitial Renal Fibrosis. Am. J. Pathol..

[18] Guerrot D., Dussaule J.-C., Mael-Ainin M., Xu-Dubois Y.-C., Rondeau E., Chatziantoniou C., Placier S. (2012). Identification of Periostin as a Critical Marker of Progression/Reversal of Hypertensive Nephropathy. PLoS ONE.

[19] Mael-Ainin M., Abed A., Conway S.J., Dussaule J.-C., Chatziantoniou C. (2014). Inhibition of Periostin Expression Protects against the Development of Renal Inflammation and Fibrosis. J. Am. Soc. Neprol..

[20] Prakoura N., Kavvadas P., Kormann R., Dussaule J.-C., Chadjichristos C.E., Chatziantoniou C. (2017). NF-κB-Induced Periostin Activates Integrin-Β3 Signaling to Promote Renal Injury in GN. J. Am. Soc. Nephrol..

[21] Satirapoj B., Tassanasorn S., Charoenpitakchai M., Supasyndh O. (2015). Periostin as a Tissue and Urinary Biomarker of Renal Injury in Type 2 Diabetes Mellitus. PLoS ONE.

[22] Hudkins K.L., Pichaiwong W., Wietecha T., Kowalewska J., Banas M.C., Spencer M.W., Mühlfeld A., Koelling M., Pippin J.W., Shankland S.J. (2010). BTBR Ob/ob Mutant Mice Model Progressive Diabetic Nephropathy. J. Am. Soc. Nephrol..

[23] Dong J., Ma Q. (2019). In Vivo Activation and Pro-Fibrotic Function of NF-κB in Fibroblastic Cells During Pulmonary Inflammation and Fibrosis Induced by Carbon Nanotubes. Front. Pharmacol..

[24] Maeda S., Matsui T., Takeuchi M., Yamagishi S. (2013). Sodium-Glucose Cotransporter 2-Mediated Oxidative Stress Augments Advanced Glycation End Products-Induced Tubular Cell Apoptosis. Diabetes Metab. Res. Rev..

[25] Ahmad M., Srinivasula S.M., Wang L., Talanian R.V., Litwack G., Fernandes-Alnemri T., Alnemri E.S. (1997). CRADD, a Novel Human Apoptotic Adaptor Molecule for Caspase-2, and FasL/Tumor Necrosis Factor Receptor-Interacting Protein RIP. Cancer Res..

[26] Jiang C., Lin X. (2012). Regulation of NF-κB by the CARD Proteins. Immunol. Rev..

[27] Liu H., Yang J., Yuan Y., Xia Z., Chen M., Xie L., Ma X., Wang J., Ouyang S., Wu Q. (2014). Regulation of Mcl-1 by Constitutive Activation of NF-κB Contributes to Cell Viability in Human Esophageal Squamous Cell Carcinoma Cells. BMC Cancer.

[28] Nakajima W., Tanaka N. (2018). The Anti-Apoptotic Protein MCL1, a Novel Target of Lung Cancer Therapy. J. Cancer Treat. Diagn..

[29] Alves M., Calegari V.C., Cunha D.A., Saad M.J.A., Velloso L.A., Rocha E.M. (2005). Increased Expression of Advanced Glycation End-Products and Their Receptor, and Activation of Nuclear Factor Kappa-B in Lacrimal Glands of Diabetic Rats. Diabetologia.

[30] Breyer M.D., Böttinger E., Brosius F.C., Coffman T.M., Harris R.C., Heilig C.W., Sharma K., AMDCC (2005). Mouse Models of Diabetic Nephropathy. J. Am. Soc. Nephrol..

[31] Tesch G.H., Nikolic-Paterson D.J. (2006). Recent Insights into Experimental Mouse Models of Diabetic Nephropathy. Nephron. Exp. Nephrol..

[32] Wantanasiri P., Satirapoj B., Charoenpitakchai M., Aramwit P. (2015). Periostin: A Novel Tissue Biomarker Correlates with Chronicity Index and Renal Function in Lupus Nephritis Patients. Lupus.

[33] Hwang J.H., Lee J.P., Kim C.T., Yang S.H., Kim J.H., An J.N., Moon K.C., Lee H., Oh Y.K., Joo K.W. (2016). Urinary Periostin Excretion Predicts Renal Outcome in IgA Nephropathy. Am. J. Nephrol..

[34] Wallace D.P., Quante M.T., Reif G.A., Nivens E., Ahmed F., Hempson S.J., Blanco G., Yamaguchi T. (2008). Periostin Induces Proliferation of Human Autosomal Dominant Polycystic Kidney Cells through AV-Integrin Receptor. Am. J. Physiol. Ren. Physiol..

[35] Satirapoj B. (2018). Tubulointerstitial Biomarkers for Diabetic Nephropathy. J. Diabetes Res..

[36] Kuhad A., Chopra K. (2009). Attenuation of Diabetic Nephropathy by Tocotrienol: Involvement of NF-κB Signaling Pathway. Life Sci..

[37] Foresto-Neto O., Albino A.H., Arias S.C.A., Faustino V.D., Zambom F.F.F., Cenedeze M.A., Elias R.M., Malheiros D.M.A.C., Camara N.O.S., Fujihara C.K. (2020). NF-κB System Is Chronically Activated and Promotes Glomerular Injury in Experimental Type 1 Diabetic Kidney Disease. Front. Physiol..

[38] Li Q., Verma I.M. (2002). NF-κB Regulation in the Immune System. Nat. Rev. Immunol..

[39] Karin M., Greten F.R. (2005). NF-κB: Linking Inflammation and Immunity to Cancer Development and Progression. Nat. Rev. Immunol..

[40] He X., Young S.-H., Schwegler-Berry D., Chisholm W.P., Fernback J.E., Ma Q. (2011). Multiwalled Carbon Nanotubes Induce a Fibrogenic Response by Stimulating Reactive Oxygen Species Production, Activating NF-κB Signaling, and Promoting Fibroblast-to-Myofibroblast Transformation. Chem. Res. Toxicol..

[41] Oeckinghaus A., Ghosh S. (2009). The NF-κB Family of Transcription Factors and Its Regulation. Cold Spring Harb. Perspect. Biol..

[42] Rybicki B.A., Sadasivan S.M., Chen Y., Kravtsov O., Palangmonthip W., Arora K., Gupta N.S., Williamson S., Bobbitt K., Chitale D.A. (2021). Growth and Differentiation Factor 15 and NF-κB Expression in Benign Prostatic Biopsies and Risk of Subsequent Prostate Cancer Detection. Cancer Med..

[43] Liang G., Song L., Chen Z., Qian Y., Xie J., Zhao L., Lin Q., Zhu G., Tan Y., Li X. (2018). Fibroblast Growth Factor 1 Ameliorates Diabetic Nephropathy by an Anti-Inflammatory Mechanism. Kidney Int..

[44] Ratnam N.M., Peterson J.M., Talbert E.E., Ladner K.J., Rajasekera P.V., Schmidt C.R., Dillhoff M.E., Swanson B.J., Haverick E., Kladney R.D. (2017). NF-κB Regulates GDF-15 to Suppress Macrophage Surveillance during Early Tumor Development. J. Clin. Investig..

[45] Wang D., Jin M., Zhao X., Zhao T., Lin W., He Z., Fan M., Jin W., Zhou J., Jin L. (2019). FGF1ΔHBS Ameliorates Chronic Kidney Disease via PI3K/AKT Mediated Suppression of Oxidative Stress and Inflammation. Cell Death Dis..

[46] Baelde H.J., Eikmans M., Doran P.P., Lappin D.W.P., de Heer E., Bruijn J.A. (2004). Gene Expression Profiling in Glomeruli from Human Kidneys with Diabetic Nephropathy. Am. J. Kidney Dis..

[47] Labrèche C., Cook D.P., Abou-Hamad J., Pascoal J., Pryce B.R., Al-Zahrani K.N., Sabourin L.A. (2021). Periostin Gene Expression in Neu-Positive Breast Cancer Cells Is Regulated by a FGFR Signaling Cross Talk with TGFβ/PI3K/AKT Pathways. Breast Cancer Res..

[48] Gillan L., Matei D., Fishman D.A., Gerbin C.S., Karlan B.Y., Chang D.D. (2002). Periostin Secreted by Epithelial Ovarian Carcinoma Is a Ligand for α_V_β_3_ and α_V_β_5_ Integrins and Promotes Cell Motility. Cancer Res..

[49] Mori S., Wu C.-Y., Yamaji S., Saegusa J., Shi B., Ma Z., Kuwabara Y., Lam K.S., Isseroff R.R., Takada Y.K. (2008). Direct Binding of Integrin α_V_β_3_ to FGF1 Plays a Role in FGF1 Signaling. J. Biol. Chem..

[50] Li G., Jin R., Norris R.A., Zhang L., Yu S., Wu F., Markwald R.R., Nanda A., Conway S.J., Smyth S.S. (2010). Periostin Mediates Vascular Smooth Muscle Cell Migration through the Integrins α_V_β_3_ and α_V_β_5_ and Focal Adhesion Kinase (FAK) Pathway. Atherosclerosis.

[51] Chen W., ten Dijke P. (2016). Immunoregulation by Members of the TGFβ Superfamily. Nat. Rev. Immunol..

[52] Wei Y., Liu S., Yu H., Zhang Y., Gao W., Cui M., Li L. (2020). The Predictive Value of Growth Differentiation Factor-15 in Recurrence of Atrial Fibrillation after Catheter Ablation. Mediat. Inflamm..

[53] Bootcov M.R., Bauskin A.R., Valenzuela S.M., Moore A.G., Bansal M., He X.Y., Zhang H.P., Donnellan M., Mahler S., Pryor K. (1997). MIC-1, a Novel Macrophage Inhibitory Cytokine, Is a Divergent Member of the TGF-β Superfamily. Proc. Natl. Acad. Sci. USA.

[54] Wischhusen J., Melero I., Fridman W.H. (2020). Growth/Differentiation Factor-15 (GDF-15): From Biomarker to Novel Targetable Immune Checkpoint. Front. Immunol..

[55] Perez-Gomez M.V., Pizarro-Sanchez S., Gracia-Iguacel C., Cano S., Cannata-Ortiz P., Sanchez-Rodriguez J., Sanz A.B., Sanchez-Niño M.D., Ortiz A. (2021). Urinary Growth Differentiation Factor-15 (GDF15) Levels as a Biomarker of Adverse Outcomes and Biopsy Findings in Chronic Kidney Disease. J. Nephrol..

[56] Carlsson A.C., Nowak C., Lind L., Östgren C.J., Nyström F.H., Sundström J., Carrero J.J., Riserus U., Ingelsson E., Fall T. (2020). Growth Differentiation Factor 15 (GDF-15) Is a Potential Biomarker of Both Diabetic Kidney Disease and Future Cardiovascular Events in Cohorts of Individuals with Type 2 Diabetes: A Proteomics Approach. Ups. J. Med. Sci..

[57] Kim Y.-I., Shin H.-W., Chun Y.-S., Park J.-W. (2018). CST3 and GDF15 Ameliorate Renal Fibrosis by Inhibiting Fibroblast Growth and Activation. Biochem. Biophys. Res. Commun..

