# Supplementary material for: Role of Periostin and Nuclear Factor-κB Interplay in the Development of Diabetic Nephropathy
Source: Cells. 2022 Jul 15;11(14):2212. doi: 10.3390/cells11142212 (PMC9320904; doi:10.3390/cells11142212)
Supplement: Supplementary file 1 [file cells-11-02212-s001.zip › cells-1774616-supplementary.pdf]

Figure S1. Survival curve of WT and ob/ob mice.

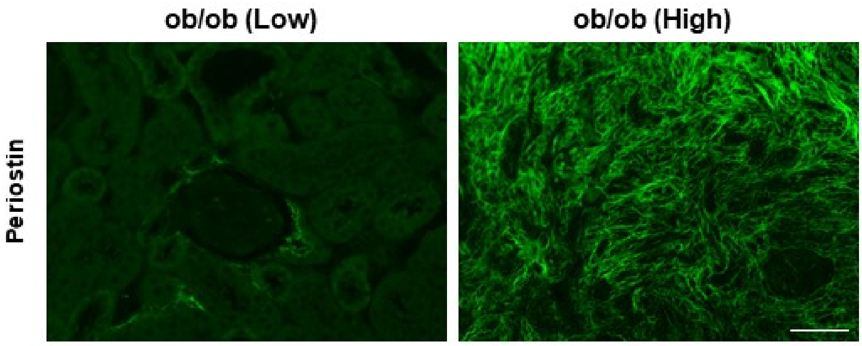

Figure S2. Periostin immunostaining in ob/ob mice without fibrosis (left x100) with fibrosis (right x200). Bars=50μm

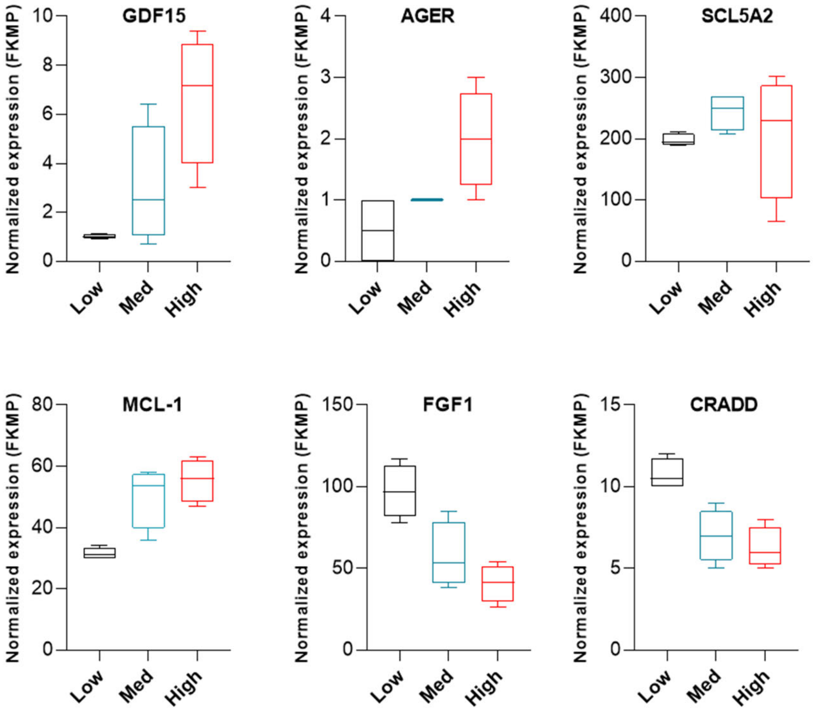

Figure S3. Regulation of FGF1, GDF15, AGER, CRADD, SCL5A2, and MCL1 with increasing periostin by RNAseq analysis.

Table S1. signalling pathways regulated in "high" periostin DN mice.

Upstream Regulator	Expr Fold Change	Molecule Type	Predicted Activation Rate	Activation z-score	p-value of overlap	Target molecules in Dataset
**NF-kB (complex)**		Complex	Activated	2,54	6,18E-03	A2M, AGER, ANXA13, C3, CALB1, CCK, CDC25B, CITED4, CRADD, DBP
**HSF1**	-1,02	Transcription regulator	Activated	2,17	7,51E-02	FGF1, FMOD, HERC4, HSPA1A/HSPA1B, Hspa1b, LPL, MCL1
**AGT**	1,43	Growth factor	Activated	2,102	1,06E-03	ACAT2, ACE, ACTA2, AGER, ATP1B1, BDKRB2, CDH13, ETV1, GSTA5, HSPA1A/HSPA1B
**AKT1**	1,052	Kinase	Activated	2,214	1,12E-02	ACAT2, ACTA2, ENO1, GDF15, GSTA5, MCL1, ME1, MYH11, NQ01, PDGFRB
**MAPK14**	1,15	Kinase	Activated	2,349	4,90E-03	ABCA1, ACTA2, CYP51A1, Defb1, GREM1, MYH11, Pmaip1, SGK1, VEGFC
**RB1**	1,155	Transcription regulator	Activated	2,414	1,00E+00	ATP1B1, CRADD, IGF1, KRT18, MET, MYOM2, SERPINE1
**NOTCH2**	1,003	Transcription regulator	Activated	2,404	1,44E-04	ACTA2, CNN1, HEYL, MYH11, RGS5, TSPAN33
**Gcg**		Other	Activated	2,236	6,40E-03	ATP1B1, GSTA5, ME1, Mup1 (includes others), SULF2
**TP53**	-1,013	Transcription regulator	Activated	2,048	5,94E-03	A2M, ACADM, ACAT1, ACE, ACER2, ACSF2, ACTA2, BCAT1, BDKRB2, CDC25B
**IL33**	1,756	Cytokine	Inhibited	-2,041	5,51E-02	ABCA1, ACAT1, LEPR, PLIN2, PRG2, SOST, TNFRSF13B, VEGFC
**ELAVL1**	-1,101	Other	Inhibited	-2,339	7,70E-03	ABCA1, ACTA2, Gvin1 (includes others), Igha, Ighv1-4, lghv1-7, Ighv14-3, Igkv8-30, JCHAIN
**Esrra**	1,039	Transcription regulator	Inhibited	-2,236	9,33E-03	ACADM, ACAT1, BDH1, ENO1, LEPR, OXCT1

Table S2. List of gene associated with NF-κB-periostin axis

Symbol	Gene Name	Location	Expr Fold Change	Expr p-value	Expr False Discovery Rate (q-value)
**ACE**	angiotensin I converting enzyme	Plasma Membrane	3,893	3,14E-04	3,46E-02
**AGER**	advanced glycosylation end-product specific receptor	Plasma Membrane	3,3	9,99E-05	2,55E-02
**APOH**	apolipoprotein H	Extracellular Space	-4,513	5,55E-04	4,12E-02
**Ces1q**	carboxylesterase 1G	Other	-14,29	3,03E-04	3,43E-02
**EPHX1**	epoxide hydrolase 1	Cytoplasm	3,262	1,81E-04	3,08E-02
**FGF1**	fibroblast growth factor 1	Extracellular Space	-2,173	3,02E-04	3,43E-02
**GDF15**	growth differentiation factor 15	Extracellular Space	3,231	1,82E-04	3,08E-02
**GREM1**	gremlin 1, DAN family BMP antagonist	Extracellular Space	29,331	3,67E-04	3,55E-02
**GST01**	glutathione S-transferase omega 1	Cytoplasm	2,905	1,96E-04	3,14E-02
**HSPA1A/HSPA1B**	heat shock protein family A (Hsp70) member 1A	Cytoplasm	2,881	3,29E-04	3,50E-02
**IGF1**	insulin like growth factor 1	Extracellular Space	-2,422	4,83E-04	3,88E-02
**KRT18**	keratin 18	Cytoplasm	2,765	3,75E-04	3,55E-02
**LEPR**	leptin receptor	Plasma Membrane	-2,459	4,73E-04	3,85E-02
**LPL**	lipoprotein lipase	Cytoplasm	-3,187	1,59E-04	2,96E-02
**INQ01**	NAD(P)H quinone dehydrogenase 1	Cytoplasm	2,243	3,45E-04	3,51E-02
**PCSK9**	proprotein convertase subtilisin/kexin type 9	Extracellular Space	-2,39	8,84E-04	4,80E-02
**PDGFRB**	platelet derived growth factor receptor beta	Plasma Membrane	-2,097	2,35E-04	3,27E-02
**PLIN2**	perilipin 2	Plasma Membrane	2,26	4,35E-04	3,74E-02
**PPARD**	peroxisome proliferator activated receptor delta	Nucleus	-2,191	3,50E-05	1,68E-02
**PROC**	protein C, inactivator of coagulation factors Va and VIIIa Extracellular Space	Extracellular Space	-2,203	2,71E-04	3,43E-02
**RBP1**	retinol binding protein 1	Extracellular Space	-5,307	2,81E-04	3,43E-02
**RXRG**	retinoid X receptor gamma	Nucleus	3,148	7,45E-04	4,55E-02
**S100A6**	S100 calcium binding protein A6	Cytoplasm	2,006	9,35E-04	4,82E-02
**UCHL1**	ubiquitin C-terminal hydrolase L1	Cytoplasm	4,91	5,91E-04	4,23E-02
